# Constraints as catalysts: a model of emotional intelligence, team learning, and team resilience, in driving creativity and innovation

**DOI:** 10.3389/fpsyg.2026.1859726

**Published:** 2026-08-06

**Authors:** Yohannes Mekonnen Yesuf, Ziska Fields

**Affiliations:** Department of Business Management, School of Management, College of Business and Economics, University of Johannesburg, Johannesburg, South Africa

**Keywords:** constraints, creativity, emotional intelligence, innovation, team learning, team resilience

## Abstract

The study explains the relationships among perceived constraints, creative solutions, and innovation team practices in tourism organizations operating in fragile, resource-scarce, and conflict-affected settings in the Amhara region, Ethiopia. Based on resilience theory and team learning theory, the research develops a complex conceptual model explaining how perceived constraints affect individual creativity and team practice in innovation via emotional intelligence, team learning, and team resilience. The study employed a cross-sectional quantitative research design and collected data via a survey administered to 287 respondents from tourism enterprises. The tourism organizations were selected using a purposive sampling method, combined with proportional and simple random sampling, from businesses located in the Amhara region. A PLS-SEM with 5,000 bootstrap resamples was used for analysis. It is found that perceived constraints do not affect both creative solution and innovation team practice. Instead, emotional intelligence fully mediates the relationship between perceived constraints and creative solutions. The mediating effect of team learning on the association between perceived constraints and team resilience is significant. The mediating effect of team resilience on the association between team learning and innovation team practices is also significant. In addition, a sequentially mediated effect of team learning and team resilience was found to be significant. This study advances the “constraints-as-enablers” perspective by demonstrating that constraints influence creativity and innovation indirectly through psychological capabilities and adaptive team processes, thereby extending resilience and team learning theories in fragile, resource-constrained tourism contexts.

## 1 Introduction

The tourism industry is among the fastest-growing globally and remains an important vehicle for socio-economic development in developing countries, including Ethiopia. In particular, tourism strongly impacts job creation, economic growth, the emergence of entrepreneurial ventures, and social exchange (Thommandru et al., 2023) service-based innovations, consumer relationships, and community involvement, and hence serves as one of the most important pillars of sustainable development. The potential of Ethiopia’s tourism sector (characterized by rich cultural history, distinctive cultural diversity, and unique natural attractions) for both internal and international markets is enormous. Nonetheless, tourism entrepreneurs are subjected to the existence of multiple and chronic constraints, namely underdeveloped infrastructure, frail institutional support, inadequate financial resources, political instabilities, and various exogenous shocks, namely climate shocks, conflicts (local) (Biru et al., 2021; Yesuf et al., 2025), etc., which jeopardize the tourism entrepreneurs’ competitive advantages and innovation capabilities.

However, on the flip side of the coin, constraints may also be an agent of innovation and creativity. The constraint-based view of creativity posits that constraints foster creativity by enabling individuals or teams to identify new ways and generate efficient responses to problems (Agarwal et al., 2021; Tromp, 2024). In Ethiopian tourism, this is evident in new forms of community-based tourism, low-cost eco-lodges, experiences that engage deeply with local culture, and the adoption of digital technology to improve customer interactions (Alamineh et al., 2023). This does not mean that all individuals or teams respond to constraints in this way. Whereas some entrepreneurs and teams succeed in leveraging limitations to create new outcomes, many do not (Shepherd and Williams, 2020). Consequently, further examination is required to understand the cognitive mechanisms, at the individual or team level, that underlie how constraints give rise to creative employees and innovative team practices.

Nevertheless, several research gaps exist, although creativity and innovation under constraints are receiving increased attention. One such gap is the overemphasis on individual-level creativity rather than on integrating team-level analysis, given that innovation in tourism businesses often involves collective resilience and learning processes. Another gap is the limited examination of the mediating role of emotional intelligence (EI) in the constraints-creativity relationship. While the positive impact of EI on coping with stress, the ability to adapt to the circumstances, and creativity has been highlighted (Carmeli, 2003; Prentice, 2020; Stoyanova-Bozhkova et al., 2022), its ability to mitigate constraints can be explained by enabling employees to manage their negative emotions in such situations, redefine constraints positively, and apply creative thinking.

Third, although team learning has been widely explored as an antecedent and outcome, it has rarely been identified as a team-level intervening mechanism that translates constraints into team resilience. In resource-constrained, turbulent environments such as those in Ethiopia, constraints can force teams to adopt learning behaviors (e.g., knowledge sharing, reflection, experimentation) to effectively translate challenges into solutions. Through this, team learning can contribute to a strong sense of collective efficacy and coping adaptability, thus building team resilience (Stoverink et al., 2020). Therefore, team learning can serve as the critical pathway to translate constraints into team resilience. Nevertheless, resilience is not built overnight; it relies on prior learning. If teams learn better, they can better establish team resilience and sustained coordination efforts in adversity, while resilient teams can reallocate resources, maintain effort, and come up with creative solutions. The sequence can be traced as constraint, team learning, team resilience, and innovation team practices (Grass et al., 2020).

Fourth, existing studies have explored creativity and innovation in stable, resource-rich contexts (Cheng and Zhang, 2020; Prayag et al., 2024). However, Ethiopia’s tourism industry is characterized by resource scarcity and weak institutions, and thus human and collective capacities seem much more important than structural resources. It can be noted that research on tourism innovation focuses mostly on facilities and policies (Elkhwesky et al., 2024), while neglecting team-level learning and resilience, which play key roles in contributing to innovation under constraints.

The study proposes a multi-level framework to explain these issues by drawing upon the constraint-based view of creativity, Salovey and Mayer’s (1990) theory of emotional intelligence, and Masten’s (2014) resilience theory. This study explains how constraints can drive both creative solutions and an innovation-oriented teamwork process in tourism organizations. It focuses on: the direct impact of constraints on creative solutions and on innovation team practices. Additionally, it considers the mediating role of emotional intelligence in the relationship between constraints and creative solutions. It considers the mediating effect of team learning between constraints and team resilience, the mediating effect of team resilience between team learning and innovation team practices, and the sequential mediation of team learning and team resilience on the relationship between constraints and innovation team practices.

Furthermore, this research contributes to the body of knowledge in three ways. Firstly, it extends the constraint-based view by providing a deeper understanding of the multi-level mediating mechanism to transfer constraints into creativity and innovation. Secondly, it enriches research on emotional intelligence by showing how it serves as a crucial mechanism linking constraints to employee creativity in a resource-constrained tourism environment. Third, it advances resilience theory by proposing that resilience is an outcome of team learning within the sequence mediation process.

## 2 Theoretical foundation and hypotheses development

### 2.1 Background theories

The study proposes a theoretically integrated model where the constraint-based view of creativity, EI theory (Salovey and Mayer, 1990), and resilience theory (Masten, 2014, 2021), are integrated to explain the transformation of constraints into creativity and innovation under a fragile and resource-limited tourism context. Instead of an aggregate of divergent individual theoretical approaches, the research model conceptualizes them as mutually interacting and contingent processes operating across different conditions. Constraint-based view of creativity serves as the core premise: environmental constraints act not only as restraints but as provocations for alternative problem-solving and resource recombination (Tromp and Sternberg, 2024). Yet, to explain mixed results on whether constraints facilitate or hinder innovation, past research tends to ignore the internal process through which the individual and team understand and manage the hardship (Mishrif and Khan, 2023).

In light of the foregoing research gap, this study conceptualizes perceived constraints as a distal contextual trigger that activates an individual’s and/or group’s psychological and collective adaptive capacity to respond to a stimulus, thereby yielding innovative outcomes. EI theory explicates an individual’s cognitive and affective appraisal process under constraints. Similar to the idea of EI as the perception, appraisal, regulation, and strategic use of emotions (Salovey and Mayer, 1990), this framework theorizes that individuals with higher levels of EI can alleviate negative affect, foster cognitive flexibility, and reframe hardship as creative opportunities. That is, EI is regarded as the primary psychological conversion mechanism transforming external constraints to creative action, going beyond prior studies that posited EI as a general predictor of an individual’s performance and leader effectiveness, to viewing EI as the adaptive capability within context, affecting an individual’s creative action under constraints (Mishra et al., 2025).

The theory of resilience and team learning theory are synthesized to account for the transformation of collective adaptation to innovative practices. This framework positions team resilience as a process-oriented, emerging, and emergent collective adaptive capacity of the team, not a fixed attribute or a distal end-state, which enables the team to cope with the disruptions, sustain functioning, and reconfigure resources under a threatening or challenging condition (Masten, 2014, 2021; Raetze et al., 2022). However, resilience is not the sole prerequisite for innovation practices; this study integrates the theory of team learning to elaborate on the conversion process that translates collective adaptive capacity into team innovation practices. Team learning process, including reflection, experimentation, integration of knowledge, and co-constructive problem solving, acts as an enabler that translates team resilience into team innovative action (Pappas et al., 2023), thus addressing prior studies that viewed resilience and learning as individual and parallel constructs.

Three important contributions to the theory of creativity and innovation in conflict-affected tourism contexts are achieved by integrating these varied perspectives into an integrated model. First, it challenges deterministic “constraint-to-innovation” theorizing by providing a contingent, process-based explanation of innovation as a product of psychological and collective adaptation (Prayag et al., 2024). Second, it moves into the domain of microfoundations research by showing how emotion capacities facilitate the transduction of negative environmental situations into creativity. Third, it extends theories on resilience and team learning to account for how adaptive team processes produce collective innovation in the face of sustained resource deprivation and disorder (Bonanno, 2021; Duah et al., 2026). Combined, the model offers a more robust account of the genesis of creativity and innovation in volatile tourism contexts, resulting from a combination of contextual pressure, emotional capacities, and the cooperative adaptation process.

### 2.2 Constraints and creative solutions

Persistent constraints in the developing and conflict-prone economies’ tourism industry (e.g., limited financial capacity, poor infrastructure, technological deficiency, institutional frailty, and market uncertainty) are evident in the tourism sector of the Ethiopian context alongside factors such as political instability, slow adaptation to the digital economy, and the lack of skilled human resources. However, these constraints force entrepreneurs and tourism firms to develop adaptive, creative responses to address these problems and remain competitive in the industry.

The link between constraint and creative response stems from a constraint-based view of creativity, which holds that constraints can facilitate divergent thinking and innovative solutions. For example, Tromp (2024) found that constraints encourage people and organizations to move beyond traditional ways and generate innovative alternative solutions. Similarly, according to Resilience Theory, adversity enhances the capability to adapt and encourages organizations to establish new forms of response to challenges. These arguments are supported by evidence from studies by Ho et al. (2023), which demonstrate that resource scarcity drives innovation in operating processes among entrepreneurs in developing countries. In addition, Korsgaard et al. (2021) found that constraints spur entrepreneurial innovation by motivating organizations to seek new opportunities and resource combinations. In the tourism industry, Utami et al. (2023) indicate that tourism entrepreneurs have generated low-cost, community-based tourism activities that utilize local culture and indigenous resources. Furthermore, Acar et al. (2019) argue that constraints increase cognitive focus and intrinsic motivation, thereby enhancing creativity. In the contemporary tourism industry (specifically in emerging economies), innovative creative solutions, such as digital platforms, community-based tourism, and resource improvisation, are key mechanisms for tackling operational and environmental constraints. Therefore, constraints may not only serve as obstacles, but also as motivators for creative action.

Therefore, the study hypothesizes that:

*H1*: Constraints positively influence creative solutions in the tourism industry.

### 2.3 Constraints and innovation team practices

Within organizations, constraints affect how teams communicate, coordinate, and innovate. The view of constraints as barriers to innovation and performance is being superseded by the view of constraints as stimuli for adaptation, learning, and innovation. This relationship can be explained by the constraint-based view of innovation, where the existence of a shortage or limitations facilitates experimentation, recombining resources, and devising new solutions (Abdelmalak, 2025). It is argued that constraints enable organizations and teams to design adaptive, rather than routine, behaviors. Resilience Theory states that adversity will lead teams to develop greater flexibility, coordination, and resilience, enabling them to navigate uncertain environments.

In the team context, constraints increase urgency and focus, prompting teams to question their routine work practices and to engage in the search for innovative ways of working. The existence of resource shortage and institutional pressure disrupts routinized work, prompting team experimentation and collaboration to achieve tasks (Shevchenko et al., 2025). Consequently, teams faced with constraints are assumed to engage in proactive, flexible, and innovative work processes. Furthermore, empirical research has identified a positive association between constraints and team innovation. Segarra-Ciprés et al. (2019) have found that constrained environments encourage innovative work practices through knowledge sharing, improvisation, and team collaboration in problem solving. Moreover, Bhaskara et al. (2023) have suggested that constraints will not directly result in innovation but rather establish conditions under which strategic team behavior can become innovative. Persistent environmental challenges can lead to the formation of innovative work practices over time through teamwork and cooperative mechanisms.

In the current context of the tourism industry, team innovation practices such as collaborative decision-making, digital coordination, improvisation, and flexible service design are crucial for organizations to adapt to a complex environment and remain competitive. In resource-constrained situations, an effective team’s adoption of constraints can lead to innovative work processes and sustained performance under uncertain conditions.

This suggests that constraints will influence the direction of work innovation.

*H2*: Constraints have a positive impact on innovation in teamwork processes.

### 2.4 The mediating role of EI

EI is defined as the ability to recognize, interpret, manage, and apply emotions in various situations effectively in guiding thought and action (Salovey and Mayer, 1990). People with elevated emotional intelligence tend to handle stress, sustain optimism, and mobilize social resources in the face of adversity (Carmeli, 2003; Liu and Boyatzis, 2021). In entrepreneurial contexts, EI enhances problem-solving, decision-making, and adaptive responses to constraints, strengthening the capacity to transform challenges into opportunities. Prior research highlights that EI is critical in reinforcing resilience by enabling individuals to regulate emotions and maintain a constructive outlook during adverse conditions (Magnano et al., 2016). Furthermore, EI acts as a buffer, reducing the negative impact of stress, suggesting that entrepreneurs with higher EI can help individuals cope more effectively with pressure and sustain resilience (Sharma and Cooper, 2016; Pathak and Goltz, 2021). In the tourism sector of developing countries, where entrepreneurs frequently confront chronic resource scarcity, political instability, and infrastructural limitations (Abbas et al., 2025; Atiase et al., 2018; Khan et al., 2020; Telfer and Sharpley, 2015), EI is a crucial mediator between constraints and creative solutions.

Entrepreneurs with higher EI are more likely to interpret constraints not as insurmountable obstacles but as opportunities for innovation. Their ability to manage emotional reactions, empathize with stakeholders, and maintain motivation allows them to channel adversity into constructive action (Page and Connell, 2020). Conversely, entrepreneurs with lower EI may experience heightened stress, diminished coping capacity, and limited creative problem-solving when faced with similar challenges (Antonopoulou, 2024; Pathak and Goltz, 2021; Sharma, 2007). Thus, EI represents an individual asset and a contextual amplifier that shapes whether constraints stimulate or suppress creative outcomes. By mediating the relationship between constraints and creativity, EI mediates the likelihood that adverse conditions will generate creative solutions rather than stagnation.

Therefore, the study hypothesizes that:

*H3*: EI positively mediates the link between constraints and creative solutions.

### 2.5 Team learning and resilience as a serial mediator

Team resilience theory posits that resilience is an emergent phenomenon developed over time through processes, rather than a one-off event (Prayag et al., 2024). Among these learning processes underlying such adaptiveness is team learning, the capacity for teams to understand their surroundings, share their knowledge, and formulate coherent responses to environmental constraints. Given the constraints in resource-limited, uncertainty-ridden environments, such as those in the tourism industry of developing countries, learning is crucial for translating constraints into innovations (Si et al., 2023).

Team learning can be conceptualized as a collaborative learning process whereby teams update their mental models and behaviors through activities such as knowledge sharing, joint reflection, soliciting and receiving feedback, and experimenting (Wallo et al., 2024). Under constraints, team members use learning processes to collaboratively make sense of problems, pool their insights, and identify potential solutions, thereby stimulating the first learning processes teams use to respond to situations rather than passively accept setbacks (Nair et al., 2024).

Over a longer period, consistent engagement in team learning processes can foster team resilience, such as by establishing shared mental models, trusting relationships, and collective efficacy, which will better help teams withstand shocks and bounce back after disruptions (Prayag and Dassanayake, 2023). Previous empirical research (e.g., Peng and Chen, 2023; Yang et al., 2025) generally supports the idea that learning-based teams are more likely to demonstrate resilient capabilities, rather than learning processes being a condition within the context.

Team resilience, in turn, helps teams implement the team adaptive capacity into an innovative team practice. When faced with pressure and the need for new solutions in a constrained context, resilient teams have greater capacity to maintain motivation to exert effort, mobilize their resource configuration, and pursue new solutions despite the persistent constraint (Dias et al., 2022; Elshaer and Saad, 2022). It could be said that team resilience is a distal outcome mechanism that translates team learning into innovative practices by mediating between team learning and those practices. Together, the study develops a serial mediation model in which constraints may lead to the development of team learning, which in turn helps develop team resilience, and then team resilience drives the generation of innovative team practices. The dynamic process indicates that innovative practices in constrained settings may not be immediate; rather, they are outcomes of a dynamic pathway of learning and adaptive capacity development.

*H4*: The relationship between constraints and team resilience will be mediated by team learning.

*H5*: The relationship between team learning and innovation team practices will be mediated by team resilience.

*H6*: Team learning and team resilience will mediate the relationship between constraints and innovation team practices.

**FIGURE 1:**
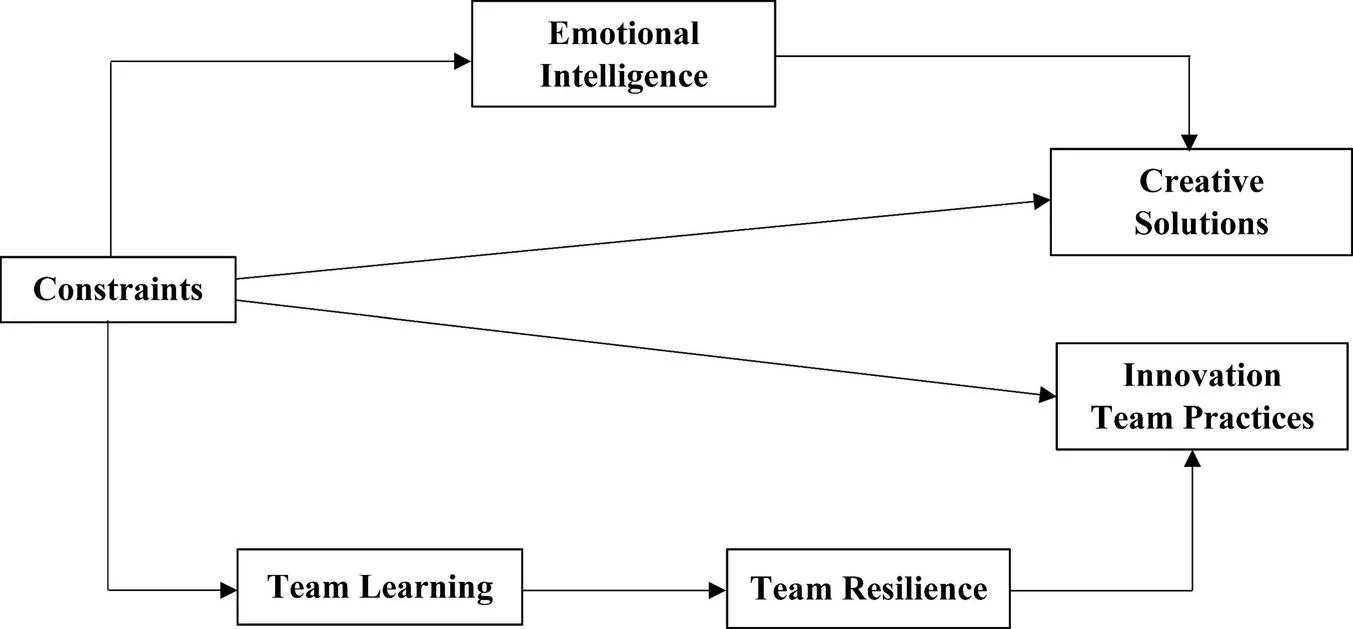
Conceptual framework.

Figure 1 presents the conceptual framework, outlining the relationships among the study variables.

## 3 Study methodology

A cross-sectional quantitative research design was applied to examine the relationships among constraints, creative solutions, and innovation team practices, considering emotional intelligence as a mediator between constraints and creative solutions, and team learning and team resilience as sequential mediators between constraints and innovation team practices.

### 3.1 Participants and procedures

Three stages of the sampling method were used. The first stage involved purposive sampling of tourism business clusters located in Bahir Dar (792 businesses), Gondar (650 businesses), and Lalibela (530 businesses) due to their high level of involvement in tourism industry activities. Bahir Dar, Gondar, and Lalibela have been chosen for their significant role in Ethiopia’s tourism sector and their relevance to the research purpose. The three cities have been chosen as major tourism destinations in the Amhara region. They offer a diverse array of attractions across cultures, history, religion, and nature; they are densely packed with tourism-related businesses, including hotels, tour operators, travel agencies, and cultural attractions. Furthermore, the selected destination has faced, and continues to face, limited resources, political instability, declining tourism numbers, poor infrastructure, etc. Thus, these cities represent suitable case studies for research on creativity and innovation under limited resource conditions. In the second stage, proportional sampling of respondents was done from each cluster (115, 95, 77). The last stage involves simple random sampling, yielding a final sample of 287 businesses from a population of 1972. The sample size was deemed sufficient for PLS-SEM, as confirmed by Monte Carlo simulations and the inverse-square-root methods recommended by Kock and Hadaya (2018). The data were gathered through personal interviews with entrepreneurs and delivered directly by the authors to ascertain the full understanding and complete answers. Entrepreneurs were intercepted in public spaces, mostly during leisure time, and asked to participate in the research voluntarily, informed that the study focused on entrepreneurs in the tourism sector and on the conditions and mechanisms of creative solutions in a constrained environment.

### 3.2 Data collection instruments

All the constructs were measured on a questionnaire, consisting of several well-established multi-item scales, specifically within an Ethiopian tourism context: the perceived constraints were measured using a 4-item scale drawn from Kozak and Martin (2012), describing barriers of financial, logistical, institutional, and socio-political nature in the context of tourism enterprises in Ethiopia (e.g., “Lack of financing resources is not limited to our business growth potential”). Team resilience was measured by the CD-RISC-10 scale developed by Campbell-Sills and Stein (2007), assessing team adaptive resources in response to change (e.g., “We can adapt to changes when they occur”). Emotional intelligence was measured by a 23-item scale developed by (Schutte et al. (1998), testing the capacity to recognize and regulate emotions in oneself and others (e.g., “I am good at reading other people’s emotions”). Individual creativity in the workplace was measured using a scale by Amabile (1988) concerning work-related creativity behaviors (e.g., “My department in this organization is creative”). Team learning was measured on a 10-item scale derived from Edmondson (1999) regarding the team’s reflection and shared learning process (e.g., “Our team regularly reflects on how effectively we are performing our work”). Finally, innovation team practices were measured on a 9-item scale widely adopted in innovation research, originally proposed by Alegre et al. (2006), about product and service improvements (e.g., “We regularly introduce innovations to our current product/service”). All the measurement instruments have been adapted in an Ethiopian tourism context.

### 3.3 Measurement

To confirm the rigor and clarity of the study, established and validated instruments were employed to measure all key constructs. Constraints were measured using the scale developed by Kozak and Martin (2012), which conceptualizes constraints as multidimensional limitations that impede strategic decision-making and innovation processes. Resilience levels were evaluated with the 10-item CD-RISC-10 (Campbell-Sills and Stein, 2007); EI was assessed using the standardized EI Questionnaire by Schutte et al. (1998); and team learning behavior was assessed using Edmondson’s (1999) method. While Creative solutions were evaluated through self-reported creativity items adapted from Amabile (1988), and innovation team practices were assessed through Alegre et al. (2006). All items were scored on a 5-point Likert scale, ranging from 1 (strong disagreement) to 5 (strong agreement). The reliability of the measures was examined using Cronbach’s alpha and composite reliability, both of which indicate internal consistency. Validity was assessed by convergent and discriminant validity to confirm that each construct was accurately and distinctly captured.

### 3.4 Analytical techniques

The study utilized Smart PLS (version 4.1.1.6) to analyze the data through PLS-SEM, a variance-based technique designed to maximize the explained variance of endogenous latent variables. PLS-SEM is particularly suitable for predictive research (Manley et al., 2021). Consistent with established standards for presenting PLS-SEM findings (Hair et al., 2017, 2021), this study adopted a structured approach to ensure methodological rigor. PLS-SEM has gained broad acceptance across management research as a valid and robust analytical tool. Several rationales underpin its use in this study. First, resilience and emotional intelligence were modeled as composites in relation to Ethiopian tourism entrepreneurs’ creativity, aligning with best practices for composite modeling in PLS-SEM (Haddoud et al., 2022). Second, the study employed constraints as predictors of entrepreneurs’ creative solutions, reflecting PLS-SEM’s strength in prediction-oriented modeling (Purwanto and Sudargini, 2021). Third, the conceptual framework exhibits a relatively complex model, encompassing multiple latent and manifest variables and multidimensional constructs (Sarstedt et al., 2021). Fourth, the link within the construct remains largely exploratory, enabling the investigation and theorization of emerging phenomena. Finally, PLS-SEM’s flexibility in assumptions and less restrictive requirements facilitated the development and estimation of the model without imposing unnecessary limitations (Hair et al., 2019).

## 4 Results

The researcher distributed 287 entrepreneur surveys, of which 235 (81.9%) were returned. However, only 219 were considered valid. To verify the aforementioned hypotheses, the study employed an SEM-PLS approach. This model consisted of three steps (Hair et al., 2021). First, the measurement model verified the reliability and validity of the study constructs, ensuring they were measured correctly. Second, the structural model was evaluated for multicollinearity, model fit, explanatory ability, and predictive relevance, and third, structural equation modeling of the associations between the variables, including indirect relationships. This allowed the study to obtain an inclusive view of the relationships between the constraints, residence, emotional intelligence, and the creative solution.

### 4.1 Assessment model

The model assessment used in the PLS-SEM analysis was tested for reliability and validity. The constructs showed satisfactory internal consistency, with composite reliability (CR) and Cronbach’s alpha (CA) exceeding the 0.70 threshold (Ringle et al., 2015). In addition, convergent validity was established, as the average variance extracted (AVE) for each construct exceeded the threshold of 0.50 (see Tables 1, 2). Moreover, discriminant validity was established through cross-loadings and the Fornell-Lacker criterion, which indicated that each item loaded higher on its corresponding construct than on any other construct (Hair et al., 2019). Tables 1, 2 shows the results for outer loadings, CA, CR, and AVE for each construct. In addition, Table 3 shows the findings of the Fornell-Lacker criterion used to test discriminant validity for each construct.

**TABLE 1:** Measurement model results of creative solutions, emotional intelligence, and innovative team practices.

Constructs	Item	Loading	Outer weights	CA	CR	AVE
Creative solutions				**0.907**	**0.913**	**0.688**
	CS1	0.825	0.201			
	CS2	0.734	0.192			
	CS3	0.767	0.177			
	CS4	0.840	0.195			
	CS5	0.915	0.230			
	CS6	0.881	0.206			
Emotional intelligence				0.958	0.961	0.635
	EI1	0.779	0.080			
	EI11	0.773	0.073			
	EI13	0.809	0.081			
	EI14	0.836	0.084			
	EI15	0.744	0.073			
	EI16	0.833	0.081			
	EI17	0.898	0.090			
	EI18	0.780	0.080			
	EI19	0.765	0.072			
	EI27	0.794	0.104			
	EI28	0.752	0.088			
	EI31	0.834	0.095			
	EI32	0.762	0.090			
	EI5	0.817	0.078			
	EI9	0.764	0.078			
Innovation team practices				0.937	0.942	0.697
	ITP1	0.878	0.167			
	ITP2	0.797	0.151			
	ITP3	0.772	0.134			
	ITP4	0.730	0.121			
	ITP5	0.926	0.153			
	ITP6	0.828	0.149			
	ITP7	0.891	0.145			
	ITP8	0.838	0.171			

Cronbach’s alpha (CA), composite reliability (CR), and average variance extracted (AVE) values represent construct-level (variable-level) results, whereas the other reported values represent individual item-level results. Therefore, all CA, CR, and AVE values are presented in bold to distinguish them from the item-level values.

**TABLE 2:** Measurement model results of perceived constraints, team learning, and team resilience.

Constructs	Item	Loading	Outer weights	CA	CR	AVE
Perceived constraints				**0.839**	**0.848**	**0.677**
	PC1	0.812	0.284			
	PC2	0.913	0.340			
	PC3	0.760	0.324			
	PC4	0.798	0.264			
Team learning				0.926	0.931	0.696
	TL1	0.883	0.193			
	TL2	0.804	0.174			
	TL3	0.758	0.155			
	TL4	0.739	0.140			
	TL5	0.915	0.182			
	TL6	0.825	0.178			
	TL7	0.896	0.170			
Team resilience				0.942	0.944	0.660
	TR1	0.796	0.108			
	TR10	0.844	0.131			
	TR2	0.861	0.132			
	TR3	0.860	0.124			
	TR4	0.717	0.110			
	TR5	0.790	0.126			
	TR6	0.754	0.125			
	TR7	0.757	0.115			
	TR8	0.827	0.119			
	TR9	0.901	0.134			

Cronbach’s alpha (CA), composite reliability (CR), and average variance extracted (AVE) values represent construct-level (variable-level) results, whereas the other reported values represent individual item-level results. Therefore, all CA, CR, and AVE values are presented in bold to distinguish them from the item-level values.

**TABLE 3:** Fornell-Larcker criterion results.

Constructs	CS	EI	ITP	PC	TL	TR
CS	0.828	0.796	0.823	0.815	0.833	0.811
EI	0.827					
ITP	0.813	0.720				
PC	0.826	0.747	0.815			
TL	0.770	0.765	0.801	0.803		
TR	0.730	0.787	0.772	0.766	0.830	

### 4.2 Structural model analysis

The model was also further assessed for multicollinearity, model fit, explanatory ability, and predictive relevance. The model was evaluated by multicollinearity using variance inflation factors (VIFs). As stated by Shrestha (2020), VIF values of 5 or lower are acceptable for multicollinearity. In this study, all VIF values were below the recommended limit (see Tables 4, 5). The model was also evaluated for fit by the standardized root mean square residual (SRMR). A value of SRMR below 0.08 is acceptable (Henseler et al., 2016); the study’s value was 0.073, indicating an acceptable model fit. The model’s explanatory ability was evaluated by the coefficient of determination (R^2^). The adjusted R^2^ values in this study were substantial and moderate: 0.859 for creative solutions, 0.748 for emotional intelligence, 0.711 for innovation team practices, 0.585 for team learning, for resilience, and 0.687 for resilience (see Table 6). The predictive relevance of this study was evaluated by Q^2^; values greater than zero indicate acceptable predictive ability.

**TABLE 4:** Outer model VIF values.

Item	VIF	Item	VIF
CS1	3.256	PC1	2.032
CS2	1.667	PC2	3.283
CS3	1.907	PC3	1.475
CS4	3.080	PC4	2.078
CS5	3.215	TL1	3.255
CS6	3.809	TL2	2.316
EI1	2.743	TL3	1.944
EI11	4.327	TL4	1.849
EI13	2.959	TL5	4.443
EI14	3.934	TL6	2.441
EI15	2.580	TL7	4.761
EI16	4.309	TR1	3.432
EI17	3.945	TR10	3.917
EI18	3.474	TR2	3.697
EI19	4.569	TR3	4.639
EI27	3.481	TR4	3.759
EI28	2.939	TR5	3.443
EI31	3.766	TR6	4.106
EI32	2.885	TR7	3.024
EI5	3.887	TR8	3.574
EI9	3.863	TR9	4.426
ITP1	3.538		
ITP2	2.355		
ITP3	2.126		
ITP4	1.915		
ITP5	4.425		
ITP6	2.623		
ITP7	4.658		
ITP8	3.479		

**TABLE 5:** VIF values for the inner model.

Item	VIF
EI - > CS	3.993
PC - > CS	3.993
PC - > EI	1.000
PC - > ITP	4.274
PC - > TL	1.000
TL - > TR	1.000
TR - > ITP	4.274

**TABLE 6:** Evaluation of model fit.

Construct	*R* ^2^	*R*^2^ adjusted	Q^2^ predict	SRMR
CS	0.860	0.859	0.648	
EI	0.749	0.748	0.747	
ITP	0.714	0.711	0.681	0.073
TL	0.587	0.585	0.763	
TR	0.689	0.687	0.755	

### 4.3 Structural equation modeling

**FIGURE 2:**
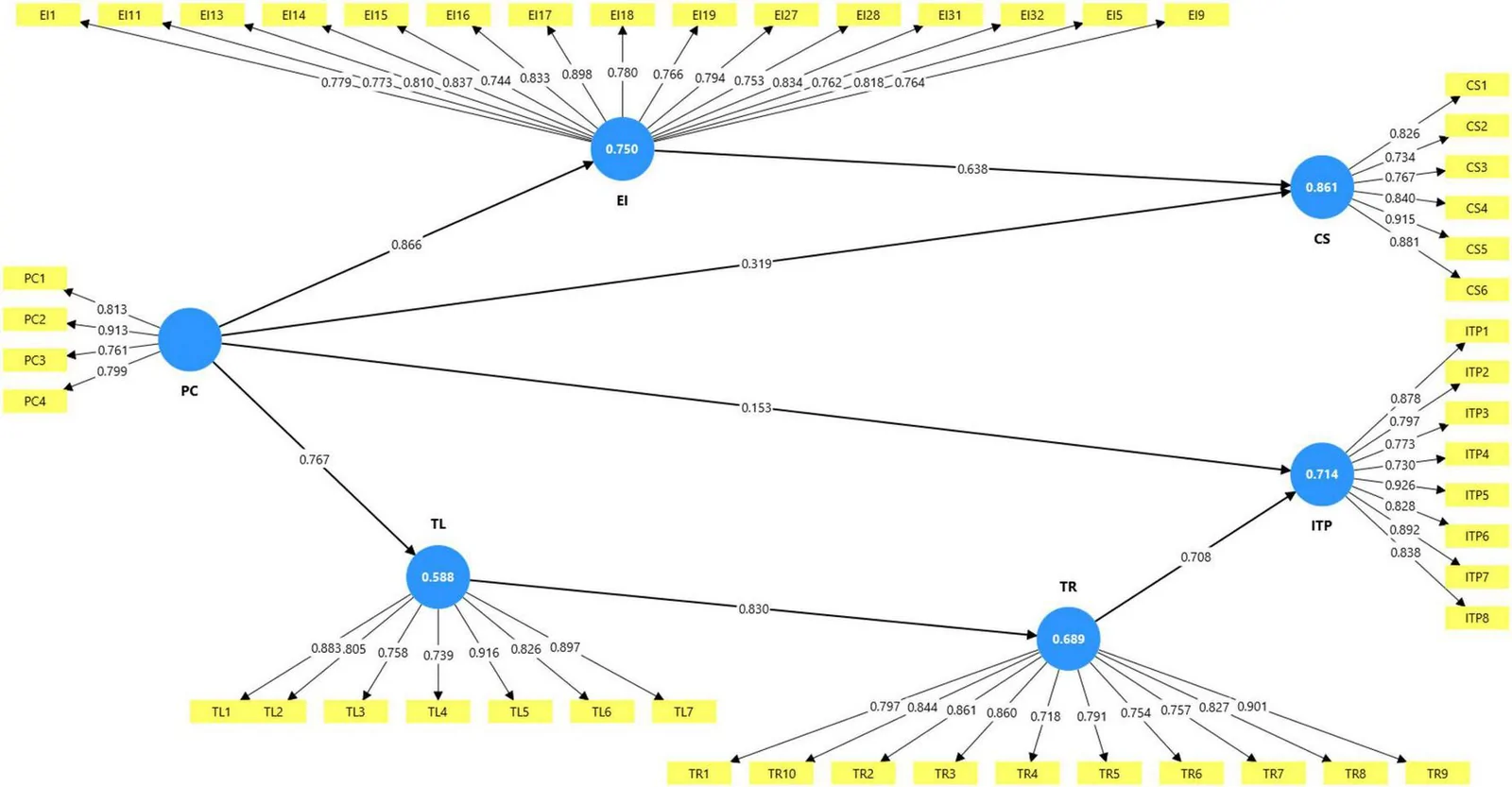
PLS-SEM path analysis.

To validate the hypotheses, the path coefficient values were examined. Significance levels were established via a bootstrapping method with 5,000 resamples. Figure 2 displays the structural model results, and Table 7 presents the path coefficients, standard deviations, t-statistics, and *p*-values. The PLS-SEM analysis showed that the hypothesized direct relationships between perceived constraints and creative solutions (H1; β = 0.319, *t* = 2.734, *p* = 0.061) and between perceived constraints and innovation team practices (H2; β = 0.153, *t* = 0.884, *p* = 0.376) were not significant. However, the mediating role of emotional intelligence on the link between perceived constraints and creative solutions is significant (β = 0.552, *t* = 8.202, *p* = 0.000), suggesting that emotional intelligence fully mediates this relationship. The mediating effect of team learning on the association between perceived constraints and team resilience (H4) is significant (β = 0.636, *t* = 12.940, *p* = 0.000), suggesting that team learning mediates this relationship. The mediating effect of team resilience on the association between team learning and innovation team practices (H5) is significant (β = 0.587, *t* = 8.060, *p* = 0.000). Furthermore, the mediating roles of team learning and team resilience in the relationship between perceived constraints and innovation team practices were significant (β = 0.450, *t* = 7.126, *p* = 0.000). Therefore, H3, H4, H5, and H6 are supported, whereas H1 and H2 were not.

**TABLE 7:** Results of path coefficients, standard deviations, t-statistics, and *p*-values.

Hypothesis	Relationships	Beta	Mean	STDEV	*t*-values	*P*-values	Decision
H1	PC - > CS	0.319	0.049	0.018	2.737	0.061	H1; not supported
H2	PC - > ITP	0.153	0.035	0.039	0.884	0.376	H2; not supported
H3	PC - > EI - > CS	0.552	0.554	0.067	8.202	0.000	H3; supported
H4	PC - > TL - > TR	0.636	0.639	0.049	12.940	0.000	H4; supported
H5	TL - > TR - > ITP	0.587	0.591	0.072	8.060	0.000	H5; supported
H6	PC - > TL - > TR - > ITP	0.450	0.455	0.063	7.126	0.000	H6; supported

## 5 Discussion

The direct effects are weakly supported, as the paths from perceived constraints to creative solutions and to innovation team practices were not significant. These findings thus indicate that perceived constraints in the present sample do not directly affect creative solutions or innovation team practices. This contrasts with the body of literature that views constraints as potential creative catalysts, enabling problem redefinition and divergent thinking at moderate levels (Cromwell, 2024; Tromp, 2024). An explanation for this result might be that in the present study; constraints are contextually extreme. In vulnerable contexts, such as conflict-affected Ethiopia, constraints may be pervasive, multidimensional, and enduring, thereby hindering rather than triggering experiments and risky behavior.

In the present approach using RBV and the contextualization perspective, we argue that the exacerbated constraints faced by tourism entrepreneurs in developing and conflict-affected regions, including financial constraints, underdeveloped infrastructure, institutional voids, and market uncertainty, often compound to create negative consequences (Trupp et al., 2025). Although a lack of resources has been directly linked to resource-combining (bricolage) and improvisational capacity, “bricolage and improvisation are contingent on basic enabling conditions” such as networks, information, and support institutions (Hernández-Barahona et al., 2023; Li and Wang, 2025), which are lacking in many conflict-affected regions. The severe structural constraints might not be translated to creative or innovative behaviors due to entrepreneurs’ limited capabilities, in these regions, in transforming them to innovative activities, because entrepreneurial activity relies heavily on external structures that are often out of the entrepreneur’s control (e.g., transportation, destinations, digital interfaces) (Hall et al., 2021).

Despite the lack of direct links, the results of the indirect links are conclusive. The mediating role of EI was found to be a full mediator in the link between perceived constraints and creative solutions, indicating that perceived constraints influence creative solutions only indirectly, through EI. Consistent with previous research, high levels of EI lead individuals to manage their negative affective experiences and stressful situations effectively and to exhibit greater flexibility in thinking under constraints, which are necessary for creative problem-solving (Stoyanova-Bozhkova et al., 2022). In tourism entrepreneurship, which operates in uncertain contexts and involves constant interaction with many stakeholders, high EI may foster adaptable coping strategies and openness to new ideas (Elshaer et al., 2024). However, because of the cross-sectional design of the current study, these mediation results must be interpreted as a linkage rather than conclusive causality.

Secondly, the constraints can have a positive relationship with the team learning process, and the team learning process can have a positive relationship with team resilience. Through the mechanism of team learning (e.g., reflection, sharing knowledge, collaborative problem solving), team members will gain a collective understanding and be better able to adapt themselves to challenges imposed by the environment (Stoverink et al., 2020), and the team learning process can then serve as a channel through which constraints impact the team’s resilience. Third, team resilience mediates the relationship between team learning and innovation team practice, implying that team learning facilitates team resilience, which in turn is positively related to innovation practices. This is consistent with the theory of resilience, which argues that the ability to absorb shocks, adapt, and persist despite adversity is an important condition for sustaining innovative activity under conditions of uncertainty (Kucuksuleymanoglu, 2025; Masten, 2021).

The final sequential mediation result provides evidence of a multilevel explanatory mechanism in which perceived constraints influence innovation team practices through the mediators of team learning and team resilience. Specifically, a review of the results suggests that constraints do not directly affect innovation practices; rather, they are channeled through an adaptive, capability-building mechanism in which constraints give rise to learning, which further enhances team resilience, enabling better innovation practices. This sequential mechanism clearly underscores the importance of both social and cognitive, group-level, capability building in resource-constrained and ambiguous circumstances. While team learning serves as a mechanism for sense-making and knowledge integration, enabling the team to reframe constraints as opportunities to experiment and solve problems, team resilience emphasizes the team’s ability to maintain function, recover from adverse events, and adapt in positive ways, thereby facilitating continued innovation. The importance of this chained mediation adds to the existing literature by illustrating that innovation within constrained situations is not merely dependent on the presence or absence of resources, but rather a product of combined social and cognitive team processes that enable transformation from constraints into capabilities. This result suggests that organizations in fragile, resource-scarce environments can enhance innovation by developing team-learning-oriented, resilient team-working environments.

## 6 Conclusion

This research sought to explore the links between perceived constraints, creative solutions, and innovation team practices among tourism enterprises in fragile, resource-scarce contexts. Using a process-based approach, it tests the commonly cited “constraints-as-enablers” paradigm, offering empirical support for the notion that perceived constraints by themselves have no statistically significant direct effect on either creative solutions or innovation team practices. This study posits that constraints alone will not directly generate innovation, especially when operating in persistently difficult environments such as conflict-affected tourism settings, where managerial attention is likely to focus on survival imperatives.

The results show that constraints operate indirectly and at psychological and collective levels. EI fully mediates the effect of perceived constraints on creative solutions, highlighting that the ability of the entrepreneur to manage emotions, think flexibly, and maintain positive affect is crucial for creativity to flourish under pressure. At the team level, the research reveals a sequential path in which perceived constraints are related to team learning, which, in turn, enhances team resilience and subsequent innovation practices. This indicates that innovation in these difficult settings is less about external pressure *per se* and more about how individuals and teams collectively perceive, learn, and adapt to adversity.

Ultimately, this study contributes to theorization by shifting attention away from direct constraint-innovation effects toward an explanation grounded in the roles of internal psychological capacities and team-based adaptive processes. This implies that constraints become productive through their interaction with internal enabling resources such as emotional intelligence, learning-oriented collaborative activities, and resilience-building interactions among team members. Henceforth, enhancing innovation in fragile tourism environments will depend more on cultivating internal and collective capacities than on relying on constraints as a source of creative energy.

## 7 Implications

The study contributes to theory by offering a complex-process perspective on how perceived constraints lead to outcomes related to creativity and innovation. Instead of a prevailing single-level or direct effects perspective, the study demonstrates how constraint effects work via combined individual- and team-level mechanisms. Individually, the study argues that emotional intelligence is a key microfoundational capability, facilitating emotion regulation and reframing constraints in creative problem-solving processes. At the team level, the step-by-step process from constraint to team learning to team resilience and finally to innovation team practices shows that it is combined and dynamic team-level capabilities that shape innovation outcomes. These analyses integrate into a complex and cohesive argument and extend the theory of resilience and the research literature on team learning by bridging both the concepts of creative solution and team innovation practices. In this view, the research explains how innovation can emerge even in fragile and constrained environments by accounting for the linkage between psychological resources and shared processes of adjustment.

The study has significant practical implications for tourism entrepreneurs, managers, and organizations working in resource-constrained or conflict-impacted environments. First, because constraints don’t directly predict innovation, organizations shouldn’t assume that providing constraints alone will trigger creativity in individuals or teams. Rather, organizations should invest in employee training to build individuals’ emotional intelligence, specifically through programs that improve self-awareness, self-management, and cognitive flexibility in interpreting and responding to adverse circumstances. Second, managers should take proactive steps to build a collaborative learning environment by promoting individual and team knowledge sharing and reflection. It’s equally important for teams to build resilience by supporting leadership, building trust, and enabling adaptive coordination, thereby helping their members keep innovating while persevering despite adversity. Thus, this research emphasizes that bolstering inherent organizational capabilities, rather than addressing an adverse condition, is central to achieving innovation in resource-limited environments.

The results underscore the importance for development organizations and policymakers to shift focus away from infrastructure and resource interventions to capabilities development interventions when engaging in tourism in fragile and conflict-affected areas. Training interventions that build psychological competencies (such as emotional intelligence) among employees and team-level capabilities (such as learning teams and resilience) need to be prioritized by policy interventions. Entrepreneurship training, mentorship programs, and knowledge-sharing platforms should be supported to enable actors to overcome the existing constraints and maintain innovation. The networking of key stakeholders within the tourism system helps to reinforce learning processes. By focusing on the development of human and social capital, policymakers can help create environments where actors are better equipped to turn constraints into opportunities for innovation, thereby ensuring long-term stability and sustainability.

## 8 Limitations and future research

This research also possesses several limitations that need to be taken into account while evaluating the results. First, the use of a cross-sectional design has precluded causal inference. The results we have found, while compatible with the proposed mediated process, remain associational in nature. The lack of temporal separation in the collection of measures means the direction of effects cannot be unequivocally attributed to a single direction. Second, because reliance is on self-report scales, concerns about common-method bias and inflated parameter estimates are valid. Although remedial actions have been taken, such as ensuring the confidentiality of responses, removing ambiguous items, and reducing apprehension during evaluation, these remedies can never completely eliminate method bias. The analysis using common-method bias diagnostic statistics (e.g., collinearity tests) indicates that neither a single latent trait nor collinearity has exceeded acceptable limits. However, it is not possible to confirm that no method variance influences the estimates using these statistical tests. As a result, the results are likely biased to some extent by the method variable. Third, the research context is specific and applies only to tourism firms located in a less developed, conflict-ridden environment. While it offers an unusual and critical examination of organizational processes under severe pressures, it may also raise concerns about the generalizability and external validity of the research findings.

From the limitations, there are several proposed future research directions: first, to assess the temporal dynamics and possible reciprocal influence among the relationships perceived constraints- emotional intelligence- team learning- team resilience- innovation practices, the use of longitudinal research designs would be necessary. Second, the developed theoretical model can be further extended by considering several other contextual and/or personality-related moderator or mediator variables (e.g., ecosystem embeddedness or leadership styles) that may explain why and when constraints positively influence creativity via individual and team psychological processes. Third, the external validity of the proposed model could be tested by conducting studies across different countries and industries and by varying the level of environmental stability. The studies could also be designed to test boundary conditions of the indirect, process-oriented impacts. Finally, researchers could use qualitative or mixed-methods approaches to explore how perceptions of constraints differ between entrepreneurs and teams. The richness this will offer can supplement micro-level process research on constraint management.

## Data Availability

The original contributions of this study are included in the article. Further inquiries can be directed to the corresponding author.
